# A Hybrid Transfer Learning Approach for Ambient PM_2.5_ Exposure Modeling in LMIC Contexts: A Case Study from Lima, Peru

**DOI:** 10.1021/acsestair.6c00125

**Published:** 2026-08-18

**Authors:** Qiang Pu, Qingyang Zhu, Elvis Medina, Alan Llacza, Bryan Vu, Jianzhao Bi, Kenan Li, Laura Nicolaou, William Checkley, Stella Hartinger, Kyle Steenland, Yang Liu

**Affiliations:** College for Public Health and Social Justice, Saint Louis University, St. Louis, Missouri 63103, United States; Gangarosa Department of Environmental Health, Rollins School of Public Health, Emory University, Atlanta, Georgia 30322, United States; National Meteorological and Hydrological Service of Peru, Lima 15072, Peru; National Meteorological and Hydrological Service of Peru, Lima 15072, Peru; Department of Environmental Health, Harvard TH Chan School of Public Health, Boston, Massachusetts 02115, United States; Department of Environmental and Occupational Health Sciences, University of Washington, Seattle, Washington 98195, United States; College for Public Health and Social Justice, Saint Louis University, St. Louis, Missouri 63103, United States; Division of Pulmonary and Critical Care, School of Medicine, Johns Hopkins University, Baltimore, Maryland 21205, United States; Department of Environmental Health and Engineering, Bloomberg School of Public Health and Center for Global Non-Communicable Disease Research and Training, School of Medicine, Johns Hopkins University, Baltimore, Maryland 21205, United States; Division of Pulmonary and Critical Care, School of Medicine, Johns Hopkins University, Baltimore, Maryland 21205, United States; Center for Global Non-Communicable Disease Research and Training, School of Medicine, Johns Hopkins University, Baltimore, Maryland 21205, United States; Centro Latino Americano de Excelencia en Cambio Climático y Salud, Universidad Peruana Cayetano Heredia, Lima 15102, Peru; Gangarosa Department of Environmental Health, Rollins School of Public Health, Emory University, Atlanta, Georgia 30322, United States; Gangarosa Department of Environmental Health, Rollins School of Public Health, Emory University, Atlanta, Georgia 30322, United States

**Keywords:** PM_2.5_, transfer learning, sampling bias, LMICs, exposure model

## Abstract

Exposure to fine particulate matter (PM_2.5_) contributes to millions of premature deaths annually worldwide. While epidemiological studies increasingly rely on geospatial technologies, such as satellite remote sensing, low-cost sensors, and chemical transport models, to quantify air pollution exposure, sparse monitoring in many low- and middle-income countries often introduces sampling bias, potentially skewing exposure estimates. We developed a hybrid and transfer learning approach in Lima, Peru, that integrates multiple geospatial technologies and machine learning to mitigate sampling bias and improve PM_2.5_ predictions. Our approach incorporated ground PM_2.5_ observations from reference-grade and low-cost monitors, satellite-retrieved aerosol optical depth, simulations from Weather Research and Forecasting model coupled with Chemistry, and additional pollution-related variables. We accounted for the sampling bias using a geo-envrionmental similarity-based transfer learning, leveraging training data from data-rich regions (the United States) to inform the data-poor target region (Lima). We then built an instance-weighted random forest model to predict daily PM_2.5_ concentrations across Lima from 2010 to 2023. The model achieved a cross-validated *R*^2^ of 0.76 and reduced potential overestimation in mountainous regions (average PM_2.5_ reduced from 34.19 to 29.23 *μ*g/m^3^). This approach provides a scalable solution to support robust epidemiological research and public health planning across data-scarce settings.

## Graphical Abstract

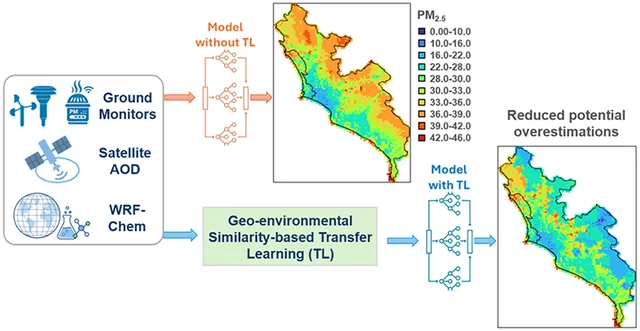

## 1. INTRODUCTION

Ambient fine particulate matter with aerodynamic diameter ≤2.5 *μ*m (PM_2.5_) remains a leading health risk factor for premature mortality and morbidity worldwide, with disproportionate exposure and disease burdens in Low- and Middle-Income Countries (LMICs).^1–3^ However, regulatory monitoring in these regions is often very limited or nonexistent because of the high cost of operation and maintenance of instruments.^4^ Significant challenges arise for accurate PM_2.5_ exposure assessment in regions with the highest pollution burdens while having the least monitoring capacity. Consequently, efforts have been largely limited regarding health effects investigation and air pollution management.^5,6^

Advances in measurement and geospatial modeling now enable the wide characterization of PM_2.5_ exposures. Low-cost sensors (LCSs) can improve monitoring coverage at relatively low expense when the sensors are well-calibrated and maintained to ensure data quality.^7–10^ For example, outdoor PurpleAir networks are widely used to supplement regulatory monitoring at neighborhood scales,^11–13^ whereas portable devices such as AirBeam support short-term campaigns and indoor/outdoor microenvironment assessment.^14,15^ Chemical transport models (CTMs), such as the Goddard Earth Observing System Chemistry model (GEOS-Chem), Weather Research and Forecast model coupled with Chemistry (WRF-Chem), Community Multiscale Air Quality Modeling System (CMAQ), and reduced-form models, can simulate formation, transport, and removal processes of air pollutants under meteorological and emissions constraints. CTMs have been used to derive spatiotemporally continuous pollutant concentration estimates, although accuracy is limited by the inherent uncertainties in emission inventories and model parametrizations.^16^ Regional applications typically have a spatial resolution on the order of a few to tens of kilometers, which may not be sufficient to capture regional spatial variability of ambient PM_2.5_.^17–19^ Satellite aerosol remote sensing provides repeated and broad observations of the atmosphere from space. Specifically, satellite-retrieved aerosol optical depth (AOD), a measure of particle loading within an atmospheric column, has been widely used to estimate surface PM_2.5_ concentrations at high spatial (e.g., 1 km) and temporal (e.g., hourly) resolutions at regional and global scales, although nonrandom data missingness caused by cloud cover and high surface reflectance remains a concern.^20–26^ In LMIC contexts with sparse monitoring, combining the complementary strengths of LCS and CTMs in a hybrid satellite-based statistical modeling framework has become pivotal for accurate PM_2.5_ exposure estimates.^8^ Such applications have been documented in a few cities in India and African countries.^5,27–30^ Nevertheless, long-term and high-resolution PM_2.5_ exposure assessment is still lacking across most LMICs.^20^

Calibrating satellite AOD to ground-level PM_2.5_ concentrations generally requires ground-monitoring data for model training and validation. The paramount issue lies in that if monitoring locations and training samples lack representation for the underlying spatiotemporal PM_2.5_ distribution, predictions of PM_2.5_ at unmonitored locations become biased and the resulting uncertainties inflate, regardless of the choice of statistical models or how well they perform in cross-validations.^31,32^ This fundamental challenge of sampling bias is particularly acute in PM_2.5_ exposure modeling in LMIC contexts where ground monitoring is rarely comprehensive and is constrained by logistical, economic, and access factors for both reference and LCS networks.^4,33,34^ Our previous study conducted in Lima, Peru, from 2010 to 2016, where the landscape and emission sources are diverse, has identified the issue of potential overestimation of PM_2.5_ concentrations over elevated areas when extrapolating a well-trained model using data collected from urban centers in lower-elevation areas (*R*^2^ = 0.70).^35^ The potential errors caused by sampling bias may ultimately propagate into the health effect estimates in epidemiological studies and may mislead policy development.

Transfer learning (TL), as a subfield of machine learning, is designed to account for differences in feature space and distribution between source (data-rich areas) and target (data-sparse areas) domains.^36^ In spatiotemporal air pollution exposure models, TL has been used to leverage data from well-monitored regions to improve predictions in data-sparse areas; examples include PM_2.5_^37–39^ and NO_2_.^40,41^ TL models used include both supervised machine learning and deep learning approaches that exploit limited amount of observational data from target areas (e.g., TrAdaBoost, bidirectional long short-term memory network, graph attention networks, Transformer-based models)^37,38,42–44^ and unsupervised TL methods (e.g., CORrelation Alignment, CORAL) that align feature distributions without ground observations in target areas,^41^ although the supervised approaches generally show higher accuracy and robustness because target-area air pollution observations provide direct constraints for model adaptation.^45^ Under the assumption that regions with high similarity geographically and environmentally may exhibit comparable air pollution patterns, high-quality and nonbiased source data can be transferred to help infer the PM_2.5_ exposure in the data-sparse target region in LMICs.^46,47^ However, transfer approaches that can reliably overcome the substantial discrepancies in pollution regimes and geographic context remain limited.

In this study, we introduce a hybrid transfer learning modeling approach to account for sampling bias arising from a sparse and environmentally unrepresentative monitoring network in Lima, Peru. Lima provides a particularly informative setting for this work because its monitoring network has limited coverage only over lower-elevation urban centers, while no routine monitoring exists across large portions of the metropolitan region at higher elevations. This work is also conducted as part of the Global Environmental and Occupational Health Hub project in Peru (GEOHealth Hub Peru, geohealthperu.org), which aims to develop high-resolution PM_2.5_ exposure estimates to support epidemiological research on the health impacts of air pollution in Peruvian populations. We integrate diverse sources of geospatial data, including satellite AOD, WRF-Chem simulations, and observations from both regulatory and PurpleAir networks, with a geo-environmental similarity-based transfer learning approach. Unlike conventional approaches that rely solely on local data, we identify geo-environmentally similar observations from the data-rich source domain in the United States and selectively transfer them to supplement our target domain of high-elevation areas of Lima, Peru, where monitors are nonexistent and the landscape is diverse. To our knowledge, this is among the first applications of transfer learning guided by geo-environmental similarity for spatiotemporal PM_2.5_ exposure modeling in a data-scarce setting in LMICs, and it demonstrates a practical strategy to account for monitoring-induced sampling bias in support of downstream epidemiological research.

## 2. DATA AND METHODS

### 2.1. Study Area and Data

Our study area covers the Lima metropolitan area, including Lima—the capital city of Peru and its adjacent port city—Callao, which has diverse topography ranging from sea level to elevations ~2700 m (see Figure 1). The Lima metro region has an estimated population of over 10 million as of 2023, making it the second largest city in South America.^48^ For model development purposes, we constructed a modeling grid with 3804 grid cells at a 1 × 1 km^2^ spatial resolution.

#### 2.1.1. Ground PM_2.5_ Observations.

The reference-grade air quality monitoring network in Lima consists of 10 stations that are Thermo *β* 5014i monitors utilizing the *β*-ray attenuation method. They are managed by the Servicio Nacional de Meteorología e Hidrología del Perú (SENAMHI), the National Meteorological and Hydrological Service of Peru. We also collected hourly PM_2.5_ measurements provided by the U.S. Embassy in Lima. These stations are concentrated in city centers, low-elevation areas and have limited representation in elevated regions. Daily PM_2.5_ concentrations from 2010 to 2023 were calculated using original hourly observations. We removed all observations that were negative or greater than 1000 *μ*g/m^3^, and data from days with less than 16 out of 24 possible measurements. As the original monitoring network is overly sparse, we also collected PM_2.5_ observations from 22 low-cost monitors of PurpleAir (https://www.purpleair.com/). We used the publicly available PurpleAir PM_2.5_ daily measurements from 2019 to 2023, although they are not fully calibrated against reference monitors. Following Bi et al.,^11^ we conducted quality control by removing daily samples with a dual-channel difference greater than 30%, and the dual-channel readings were averaged to provide PM_2.5_ measurements. We also excluded readings with daily PM_2.5_ concentrations greater than 1000 *μ*g/m^3^ and those with missing or abnormal temperature and humidity (humidity should be within 0 to 100% and temperature should be within −20 to 140 °F). This process removed about 17% of the raw PurpleAir observations and retained 4023 daily observations in total. Additionally, we included 2081 daily observations from 2011 to 2013 derived from our previous study,^35^ which are based on measurements from 15 mobile research-grade calibrated monitors operated by Johns Hopkins University (JHU stations).^49^ We then integrated the three sets of observational data into the 1 km^2^ modeling grid. We calculated averaged values if multiple stations fell within a single grid cell and used only SENAMHI data if SENAMHI and PurpleAir stations fell in the same grid cell. As a result, our monitoring data cover a total of 30 1 km^2^ grid cells as modeling locations, with 11 based on reference-grade stations (SENAMHI and U.S. Embassy), 13 from PurpleAir, and 6 from JHU sites (Figure 1).

#### 2.1.2. Satellite AOD Data.

We used the satellite-retrieved AOD data from the Moderate Resolution Imaging Spectroradiometer (MODIS) aboard satellites of Terra and Aqua using Multi-Angle Implementation of Atmospheric Correction (MAIAC) algorithm between 2010 and 2023.^50^ The MAIAC AOD provides global daily aerosol measurements at 1 km^2^ spatial resolution with two equator crossing times at about 10:30 a.m. and 1:30 p.m. local solar time, respectively. MAIAC is based on time series image processing and considers the effects of bidirectional surface reflectivity, which effectively retrieves AOD over both bright and dark vegetated surfaces.^50,51^ Numerous validation studies have consistently reported high data accuracy of MAIAC AOD.^52,53^ We used the MCD19A2 (at 0.55 *μ*m) combined Terra/Aqua aerosol product under MODIS Collection-6.1 data record. To ensure the quality of MAIAC AOD data, we used AOD values only with the corresponding quality assessment flag of *best quality*. We also gap-filled the daily AOD values using the tree-based imputation method in Bi et al.^54^ and Pu and Yoo.^55^ Missing AOD values were imputed using six aerosols reanalysis products from the Modern-Era Retrospective analysis for Research and Applications, Version 2 (MERRA-2), including total AOD, black carbon, dust, organic carbon, sea salt, sulfate from *M2T1NXAER*, together with meteorological variables from WRF-Chem (described in Section 2.1.3). All variables were aggregated at daily level and were resampled at 1 × 1 km^2^ resolution using inverse distance weighting. Detailed imputation processes can be found in Sec. 3.2.1 in Pu and Yoo.^55^

#### 2.1.3. Chemical Transport Model (CTM) Simulations.

We simulated PM_2.5_ concentrations and a range of geophysical variables related to the dispersion and formation of PM_2.5_ from 2010 to 2023 at a 5 × 5 km^2^ resolution using the WRF-Chem model. WRF-Chem is widely used for simulating regional air quality worldwide,^56^ and it has been adopted as the operational air quality-forecasting platform by SENAMHI. Initial and boundary meteorological conditions were from ERA5, the fifth generation reanalysis from ECMWF (European Centre for Medium-Range Weather Forecasts). However, due to lack of industrial and biogenic emissions, we only considered a vehicular emission model developed by the IAG-USP Laboratory of Atmospheric Processes, which may lead to increased uncertainty of simulated PM_2.5_. See detailed model configurations in Sánchez-Ccoyllo et al.^57^ Following our previous study conducted in Lima,^35^ this study used the surface-level hourly WRF-Chem simulation outputs, including PM_2.5_ dry mass concentration, meteorological variables (cloud cover, surface pressure, air temperature, u- and v-wind components, and planetary boundary layer height), and surface conditions (albedo, vegetation cover, downward short-wave flux at surface, accumulated/upward heat flux at the surface, soil temperature, soil moisture). Hourly WRF-Chem data were then aggregated at a daily level and were interpolated onto the 1 km^2^ modeling grid using inverse distance weighting.

#### 2.1.4. Auxiliary Variables.

We included a wide range of auxiliary variables that are related to surface geographic conditions and PM_2.5_ emissions. We collected annual land cover data from 2010 to 2020 at 300 m resolution from ESA Land Cover Climate Change Initiative.^58^ We calculated the land cover percentage in our 1 km^2^ grid and used data from 2020 to estimate that for years from 2021 to 2023, given that land cover change after COVID appeared insignificant spatially.^59^ Our land cover types include build-up, bare land, forest, shrub, grass, crop, wetland, and water. We also integrated the annual gridded population count data for 2010–2023 at a 30-arc second resolution (~1 km) from LandScan (https://landscan.ornl.gov/). Annual road network data from 2010 to 2023 was downloaded from Open-StreetMap via Geofabrik (https://www.geofabrik.de/). For each grid cell, we calculated total road length of road types for motorized vehicles. We also calculated grid-level mean elevation using the DEM data at 30-arc second resolution (~1 km) from the ASTER Global Digital Elevation Model V003 (10.5067/ASTER/ASTGTM.003).

### 2.2. A Geo-Environmental Similarity-Based Transfer Learning Approach

We develop a geo-environmental similarity-based TL approach that selectively borrows information from a source domain (well-monitored high-elevation areas in the United States such as California, Colorado Plateau) to improve prediction over the elevated areas of the target domain, namely, Lima Metro (no monitoring >375 m above sea level) (simplified workflow shown in Figure 2). The key is to transfer only from source samples whose geographic-environmental context closely matches that of the target locations, while accounting for their discrepancy in domain distributions.^60^ Our implementation here combines dimension reduction, clustering, similarity quantification, and domain adaptation, although alternative heuristics can be adopted.

#### 2.2.1. Similarity-Based Source Selection.

To quantify geo-environmental similarity, we first reduce the dimensionality of geo-environmental covariates, including meteorological variables, elevation, land cover types, and satellite AOD, using principal component analysis, PCA.^11^ Covariates are standardized using target moments, and we retain PC components explaining ≥75% of variance. Second, to characterize Lima’s high-elevation zones, we structure high-elevation grid cells into compact environmental neighborhoods by clustering in the PCA space with the density-based spatial clustering of applications with noise (DBSCAN), specifically, a *k*-nearest neighbor (*k*NN)-guided DBSCAN was used for its efficiency and robustness to noise.^61,62^ The *k*NN-DBSCAN builds clusters from a *k*NN graph rather than exhaustive *ε*-range searches (*ε* chosen from the *k*NN distance curve) and preserves cluster structure while dramatically reducing neighbor-search cost with improved scalability in our PCA space.^61^ We set *k* ∈ {4, 6, 8, 10,12,16} (with 8 PCs) and *k* = 8 yielded stable clusters and was used in the main analysis. Third, for each day, we quantify cluster-based geo-environmental similarity between source samples (high-altitude U.S.) and target neighborhoods by computing a Mahalanobis distance in the PCA space. The distance is mapped into a scalar similarity score in (0,1], which is used to rank geo-environmental similar source points. We retain the top 1% fraction to form transfer set, denoted as *S*_trans_, which consists of daily PM_2.5_ observations along with covariates from the United States that are most geo-environmentally similar to high-elevation Lima. The choice of 1% fraction here is to balance sample representativeness against undue influence of transferred sample.^11^

#### 2.2.2. Domain Adaptation and Weighting.

Unlike simple data merging, we apply a trained TrAdaBoostR2 model to the selected transfer set *S*_trans_ to account for distributional differences between the source (U.S.) and target (Lima) domains. The model was trained using low-elevation data from both source and target domains, allowing it to learn the distribution shift between them. We use TrAdaBoostR2 given its model performance and robustness to avoid negative transfer,^38^ compared with alternative models, such as CORrelation ALignment (CORAL).^41^ To differentiate the relative importance of transferred samples from local samples, we assign each transferred sample point *s* a weight of *ω*_s_ (equal to scalar similarity score, see above) and unit weight for target samples. The domain-adapted transfer samples were subsequently combined with local Lima observations to construct the final model-training data set. The resulting augmented and similarity-weighted training data set can be represented as

$${𝒟}_{\text{train}}={\left\{\left(y^{\text{T}},X^{\text{T}},1\right)\right\}}_{\text{target}}\cup {\left\{\left(y^{\text{s}},X^{\text{s}},\omega_{\text{s}}\right)\right\}}_{s\in S_{\text{trans}}}$$

where *y*^T^ represents the daily PM_2.5_ observations in Lima, *y*^s^ is the domain-adapted PM_2.5_ concentrations using TrAdaBoostR2, and ***X*** denotes a vector of various covariates from the target domain (Lima; ***X***^T^) and the source domain (U.S.; ***X***^s^).

### 2.3. Ambient PM_2.5_ Predicting Model for Lima

Finally, given 𝒟_train_, we use Random Forest (RF), a bagging-based ensemble of decision trees that has been extensively used in modeling ambient PM_2.5_ concentrations to predict daily PM_2.5_.^23,24^ We fitted an instance-weighted RF model, denoted as ${\hat{f}}_{\text{RF}}=\text{RF}\left({𝒟}_{\text{train}}\right)$, where sample weights are from 𝒟_train_ and hereafter referred to as TL_RF.

The prediction of PM_2.5_ concentrations, ${\hat{Y}}_{i,j}$, at grid cell *i* and date *j* in Lima is then:

(1) $${\hat{Y}}_{i,j}={\hat{f}}_{\text{RF}}\left(X_{i,j},Z_{i}\right)$$

where ***X***_*i*,*j*_ represents the vector of all spatiotemporal covariates in Lima, comprising daily variables of gap-filled MAIAC AOD, WRF-Chem simulated PM_2.5_ along with meteorological factors (downward short-wave radiation and planetary boundary layer height, cloud fraction, air temperature at 2 m, surface air pressure, wind components at 10 m), annual variables of total road length, gridded population count, and eight land cover types at grid cell *i* and date *j*. *Z*_*i*_ is the time-invariant covariate of elevation at grid cell *i*. Because regional health data are reported at the district level (43 in Lima city and 6 in Callao), we also calculate the population-weighted PM_2.5_ concentrations for each district to investigate the potential impact of incorporating transfer learning approach in exposure assessment (see in the SI).

We employed a 10-fold cross-validation (CV) technique for both model development and validation.^63^ The data set was randomly divided into 10 subsets with nine used as the training set and the remaining one as the testing set. This process was repeated 10 times, until each subset was used as the testing set. For model development, 10-fold CV was adopted for tuning the values of hyperparameters for the RF model (Table S1). To assess cross-data set consistency given that PurpleAir observations were not calibrated against reference monitors, we conducted an out-of-sample consistency check by evaluating predictions from an otherwise identical model trained without using PurpleAir data. Final predictions, however, were generated from the model trained on the full data set. We evaluated model performance using the metrics of goodness-of-fit (*R*^2^) and root-mean-square error (RMSE). We used Shapley additive explanations (SHAP), specifically TreeSHAP, and summarized variable contributions by mean absolute SHAP values. SHAP provides a game-theoretic, model-agnostic framework that has been widely used for interpreting the feature effects. See theoretical and implementation details in ref 64. We used R software (version 4.3.2) for model development and SHAP (0.43.0, Python 3.12) for model interpretation.

It is important to note that direct empirical validation in high-elevation Lima is not feasible because of the lack of ground monitoring. Consequently, model performance in these regions was assessed indirectly through consistency with domain knowledge and comparison with emission-driven simulations from WRF-Chem and GAINS (greenhouse gas–air pollution interactions and synergies).

## 3. RESULTS

### 3.1. Description of Ground-Based PM_2.5_ Measurements

Upon integration within the 1 km^2^ modeling grid, we retained a total of 26,713 daily PM_2.5_ ground observations in Lima from 2010 to 2023 for modeling, with 20,609 from the reference-grade stations (18,916 from SENAMHI and 1,693 from U.S. Embassy), 2,081 from JHU stations, and 4,023 from the PurpleAir network. The overall temporal data coverage is 29.7% of all potential monitoring-days, implying substantial data missingness (70.3%) and imbalanced data availability across space and time (Figure S1). We note that the diverse terrain in Lima Metro is poorly represented by the existing monitoring networks, particularly high-elevation areas. All current monitors are concentrated in low-elevation urban areas, with elevation between 25 and 375 m above sea level, whereas the elevation of metropolitan area spans 0 to 2,727 m (with a mean of 585 m andand SD of 505 m) (see Figure 1). SENAMHI stations recorded slightly higher concentrations and more severe pollution episodes than JHU and PurpleAir stations. A likely cause could be the different siting locations, where the nonreference grade sites are clustered near the coastal city center and have intermittent sensor operation, and prevailing onshore winds generally lowered local PM_2.5_concentrations.^35^ Another cause could be due to their differences in measurement monitors/techniques. The mean daily PM_2.5_ concentration was 27.2 *μ*g/m^3^ (SD of 12.9, range 1.6–157.8 *μ*g/m^3^) for SENAMHI, 18.90 *μ*g/m^3^ (SD of 4.7, range 9.8–41.7 *μ*g/m^3^) for JHU, and 21.2 *μ*g/m^3^ (SD of 9.9, range 0–68.0 *μ*g/m^3^) for PurpleAir. On average, annual PM_2.5_ from all networks, overall mean of 22.2 *μ*g/m^3^ and SD of 13.9 *μ*g/m^3^, exceed the WHO annual guideline (5 *μ*g/m^3^) over four times, which underscores persistent air quality challenges in Lima.

### 3.2. Transferred Samples: Selection and Representativeness

We obtained 6,552 geo-environmental similar and high-elevation samples from the United States, comprising 19.6% of the final model training data set and helping alleviate Lima’s monitoring gap in areas 375 m or higher above sea level. Transferred samples were from 340 unique source locations spanning a broad elevational range from 370 to 2658 m, with an average of 546 samples per month (SD of 70.8) to ensure balanced spatial and temporal coverages. Then, a domain adaptation of TrAdaBoostR2 was conducted to address differences in domain distributions. We also compared TrAdaBoostR2 against common domain adaptation model of CORAL using low-elevation Lima data for testing (Figure S2). CORAL, due to its unsupervised nature, performed poorly (*R*^2^ = 0.03), with pronounced underestimates at low PM_2.5_ levels and overestimates at high levels. In contrast, TrAdaBoostR2 achieved strong calibration, with an *R*^2^ of 0.64, with the majority of predictions clustering around the 1–1 line and markedly reduced transfer prediction error. With domain adaptation implemented, the daily average PM_2.5_ concentration of transferred samples was 7.5 *μ*g/m^3^ (SD of 7.8 *μ*g/m^3^), consistent with typical pollution conditions simulated in elevated regions of Lima (Figure S2).

### 3.3. Model Performance and Long-Term PM_2.5_ Predictions

Using the augmented training data set, the daily PM_2.5_ prediction model, namely, TL_RF, was built for Lima from 2010 to 2023. TL_RF achieved high prediction performance as evidenced by a 10-fold CV-*R*^2^ of 0.76 and RMSE of 6.85 *μ*g/m^3^. Figure 3a shows strong agreements between the observed and predicted PM_2.5_ concentrations during the 10-fold CV, with some underestimates during highly polluted days. We also found that our model typically underperforms for stations and days with very sparse and discontinuous observations. Variable importance computed using SHAP shows that planetary boundary layer height (PBLH) and WRF-Chem simulated PM_2.5_ are the most influential predictors, followed by meteorological variables and population (Figure 3b). Satellite-based MAIAC AOD and land cover and emissions proxies, such as bare land, built-up area and road length, also ranked highly. Geophysical radiative terms (downward short-wave flux at surface, SWDownward) and near-surface winds have smaller but non-negligible contributions. Despite the high importance of WRF-Chem simulation, its standalone performance against reference-grade observations was limited (daily *R*^2^ = 0.06, RMSE = 27.9 *μ*g/m^3^; annual *R*^2^ = 0.17, RMSE = 22.1 *μ*g/m^3^, Figure S3). Overall, WRF-Chem provides physically based information on emissions, atmospheric transport, and chemistry that helps capture broad spatial variability in ambient PM_2.5_ concentrations, while other predictors with temporal variability (e.g., AOD and meteorological variables) jointly provide complementary information that contributes to improved overall model performance. The pattern is physically coherent with the previous literature, where meteorological and geophysical terms capture stability and mixing potential of ambient PM_2.5_, whereas AOD and land cover terms encode emission intensity. For example, low PBLH values that represent shallow mixing (blue dots, Figure 3b) tend to lead to higher PM_2.5_ predictions, consistent with reduced dilution and increased pollutant accumulation.^35,54,65,66^ Besides, we find our model predictions agreed well with PurpleAir observed PM_2.5_, with an *R*^2^ of 0.54, and RMSE of 7.62, indicating consistent model performances across multiple observation networks.

Figures 4 and S4 show the annual PM_2.5_ distributions predicted by TL_RF for years from 2010 to 2023. We observed a relatively stable spatial pattern with clear interannual variability. Spatially, the highest long-term pollution levels persistedpersisted along the central urban corridor and major transportation and industrial belts (~25–41 *μ*g/m^3^ on average), while PM_2.5_ concentrations accumulated along interior valleys and gradually declined toward the Andean foothills and mountainous regions (~14–29 *μ*g/m^3^ on average). These spatial patterns may be influenced by prevailing coastal sea breezes from the south and southwest that ventilated the coast, transported pollutants inland, and were partially trapped against the steep topography of Andes. The most affluent and populated coastal districts in Lima have relatively lower pollution levels (~16–27 *μ*g/m^3^ on average) (Figures S4 and S5). For example, overall PM_2.5_ in affluent areas (top 5% by estimated GDP-PPP, gross domestic product based on purchasing power parity^67^) was 11% lower than less-affluent outskirts (GDP-PPP below 75th percentile). Temporally, average predicted PM_2.5_ levels across Lima were relatively high in 2010 (23.3 *μ*g/m^3^), dipped during 2011–2013 (~21.5 *μ*g/m^3^), then rose steadily from 2014, peaking in 2018 (25.0 *μ*g/m^3^). A notable transient dip is evident in 2020 (22.8 *μ*g/m^3^), reflecting the effects of COVID-19 mobility restrictions, followed by a rebound in 2021–2022 (mean PM_2.5_ of 24.3 *μ*g/m^3^). The reduction was most pronounced over the trafficinfluenced central urban corridor and was insignificant over coastal and elevated interiors. Despite a declining trend since 2018, annual PM_2.5_ for most urban districts remain ≥5 times above the WHO guideline (5 *μ*g/m^3^), underscoring a sustained air-quality challenge in Lima.

### 3.4. Impacts of Transfer Learning on PM_2.5_ Predictions

We assessed the impacts of transfer learning on resulting predictions by contrasting TL_RF with a baseline RF model without TL (namely, Regular_RF) that used identical hyperparameters. Although demonstrated nearly identical performance against “ground-truth” of SENAMHI data in 10-fold CV (*R*^2^ of 0.70 and 0.72, respectively, for Regular_RF and TL_RF), notable differences were found in their spatial predictions. We noticed an inflated estimate of 12.7 *μ*g/m^3^ of Regular_RF on average, most of which were found along interior elevated areas and the Andes mountain systems, with negligible changes along the coast and central urban areas (Figures 5 and S6). When comparing the model predictions from both models over elevated areas (≥370 m), TL_RF yielded substantially lower predictions than that of Regular_RF, with means of 22.6 *μ*g/m^3^ (SD of 5.7) and 42.8 *μ*g/m^3^ (SD of 4.8), respectively (Figure 5b). However, due to the absence of high-elvation monitoring data in Lima, direct validation of model prediction in these areas is currently not possible. As an indirect comparison, we evaluated the spatial patterns of TL_RF predictions against simulations from WRF-Chem and GAINS. As shown in Figures S3, S4, and S7, pollution distributions from all three modeling approaches exhibited similar spatial gradients, with lower PM_2.5_ concentrations in elevated regions (Eastern half of the Lima metro) relative to the urban core (Central West).

We also explored impacts of TL on the population-weighted PM_2.5_ concentrations at district level. During our study period, the average population-weighted PM_2.5_ concentration was 23.0 *μ*g/m^3^ from TL_RF (IQR of 7.0), compared with 25.1 *μ*g/m^3^ (IQR of 9.1) from Regular_RF. Overall overestimation at district-level ranges from 0.28 to 15.76 *μ*g/m^3^ without accounting for sampling bias. As shown in Figure S8, the highest levels of inflated estimates (Regular_RF–TL_RF) were found for districts in the central-west part of Lima, namely, Chaclacayo, Cieneguilla, Lurigancho, and Pachacamac, with differences of 15.8, 15.7, 10.9, and 9.6 *μ*g/m^3^, respectively. Bias of this magnitude could be epidemiologically relevant in principle; however, because these districts of Lima are scarcely populated, any resulting changes in health effects estimates are likely to be minimal.^68,69^

## 4. DISCUSSION

In this study, we proposed a hybrid transfer learning approach for modeling ambient PM_2.5_ in LMIC settings, and we demonstrated the effectiveness of our approach by modeling daily PM_2.5_ concentrations for Lima Metro, a setting characterized by sparse monitoring, complex topography, and heterogeneous pollution conditions. By transferring geo-environmentally similar observations from data-rich regions to data-poor regions, we aimed to account for sampling bias that arises when monitors are preferentially located in certain environmental settings (e.g., low-elevation urban centers). The underlying hypothesis is that including transferred observations from comparable geo-environmental conditions (e.g., elevated and sparsely vegetated areas) should improve model extrapolation to those unsampled regions, leveraging existing resources and infrastructure from data-rich domains. As shown in Section 3.4, the transfer learning-based model, TL_RF, produced substantially lower PM_2.5_ predictions in high-elevation regions compared to Regular_RF (without TL), highlighting important differences in how the models extrapolate to unmonitored areas (Figure S6). We posit that the TL_RF predictions are more physically plausible in these unmonitored elevated areas, given the consistency of its predicted spatial gradients with both WRF-Chem and GAINS simulations. Elevated regions in Lima are typically farther from major emission sources concentrated in the low-elevation urban valley and industrial zones, where atmospheric dispersion processes generally lead to lower pollutant concentrations with increasing elevation and distance from sources.^57,70,71^ While agreement among models does not constitute independent validation, it provides supporting evidence that the proposed transfer learning approach produces predictions consistent with established atmospheric chemistry and emission-based modeling systems (Figures S3, S4, and S7). In validation against ground-based reference-grade observations, our model achieved an annual *R*^2^ of 0.88, substantially higher than 0.52 using predictions from a global model that covers Lima Metro.^72^ This indicates improved agreement with ground-truth observations, reflecting the reduction of local sampling bias via the TL. Nevertheless, the lack of ground-truth monitoring in Lima’s elevated, unmonitored areas means that this improvement remains an inference based on domain knowledge and atmospheric science principles, rather than empirical validation. Despite this limitation, our approach provides a feasible solution for enhancing PM_2.5_ modeling by correcting sampling bias in LMICs, where expanding ground monitoring networks may be financially or logistically constrained.

Although various transfer learning approaches, particularly deep learning-based models, have been introduced in recent years,^37,39,41^ their applications in LMIC settings remain limited except for prior studies conducted in Lima.^38,45^ Again, a key barrier to broader implementation is the scarcity of ground monitoring data, adequate predictor variables, and computational infrastructure, which constrain the feasibility of highly complex modeling frameworks in many LMIC contexts. In this study, we prioritized robustness and scalability over the algorithmic complexity. By leveraging widely available satellite-derived data products (e.g., MAIAC AOD) and computationally efficient algorithms (e.g., PCA, TrAdaBoostR2, and Random Forest), our approach operationalizes transfer learning in a manner that is both data-efficient and broadly deployable. Such a design enhances the scalability and reproducibility of PM_2.5_ exposure modeling across diverse LMIC regions.

Our study also has direct relevance to environmental and epidemiological research. Correcting potential overestimation over unmonitored areas may reduce the level of exposure misclassification. More accurate and credible spatiotemporal predictions are needed to better inform future monitoring network expansion and facilitate pollution management and control policy development. The GEOHealth Hub Peru, geohealthperu.org, has established a low-cost sensor air quality monitoring network across 176 locations nationwide using PurpleAir.^8^ Our proposed approach can be readily integrated with the monitoring network for modeling of nationwide PM_2.5_ exposure levels.

Several limitations of this study warrant emphasis. First, the geo-environmental similarity metric used, Mahalanobis distance in PCA space, may be insufficient when multiple atmospheric regimes are mixed or relationships are nonlinear. Emerging spatiotemporal contexts learning approaches, such as Google’s AlphaEarth, could provide a more robust way to quantify geo-environmental similarity by leveraging high-dimensional latent representations trained on billions of satellite imageries across millions of locations globally.^73^ However, the underlying assumption that locations with similar geo-environmental conditions will have similar pollution levels may oversimplify the pollutant evolution; thus, the transferred samples may add bias to the final predictions. Similarly, the absence of ground monitoring data in elevated areas of Lima limits direct empirical validation of our model. Strategic deployment of a small number of reference-grade or calibrated low-cost monitors, e.g., PurpleAir, in these regions would substantially strengthen the evaluation of similarity-based transfer learning. Finally, in modeling the ambient PM_2.5_ concentration in Lima, we did not explicitly account for the impacts of extreme pollution episodes, such as severe stagnation unique to Lima, which may contribute to mild underestimation at high concentrations. Continued expansion and calibration of the PurpleAir network could improve event capturing.

## 5. CONCLUSIONS

We introduced a geo-environmental similarity-based transfer learning approach for addressing spatial sampling bias in ambient PM_2.5_ modeling, using Lima as a case study representative of LMIC contexts. We first conducted daily level similarity quantification between high-elevation areas in a target domain with sparse data (Lima) and a source domain (United States). The most geo-environmentally similar samples from the source domain (data-rich) were then transferred to supplement training data for unmonitored/elevated areas in Lima. We then constructed an instance-weighted random forest model for predicting daily PM_2.5_ concentrations in Lima from 2010 to 2023. Our approach produced substantially lower PM_2.5_ predictions in unmonitored elevated areas compared to conventional modeling, with spatial patterns more consistent with atmospheric dispersion principles and process-based simulations while preserving prediction accuracy in areas where ground-truth data exist. The resulting exposure estimates will enable downstream epidemiological investigations in this understudied region. We also believe that our approach is flexible and scalable, offering a practical PM_2.5_ modeling approach for other LMIC regions facing sparse and uneven monitoring.

## Supplementary Material

Supporting Information

The Supporting Information is available free of charge at https://pubs.acs.org/doi/10.1021/acsestair.6c00125.

An additional section provides further methodological details; and additional figures and tables present supplementary results, further evaluation of the analytical approach, model performance comparisons, and indirect validation of model predictions (PDF)

## Figures and Tables

**Figure 1.**
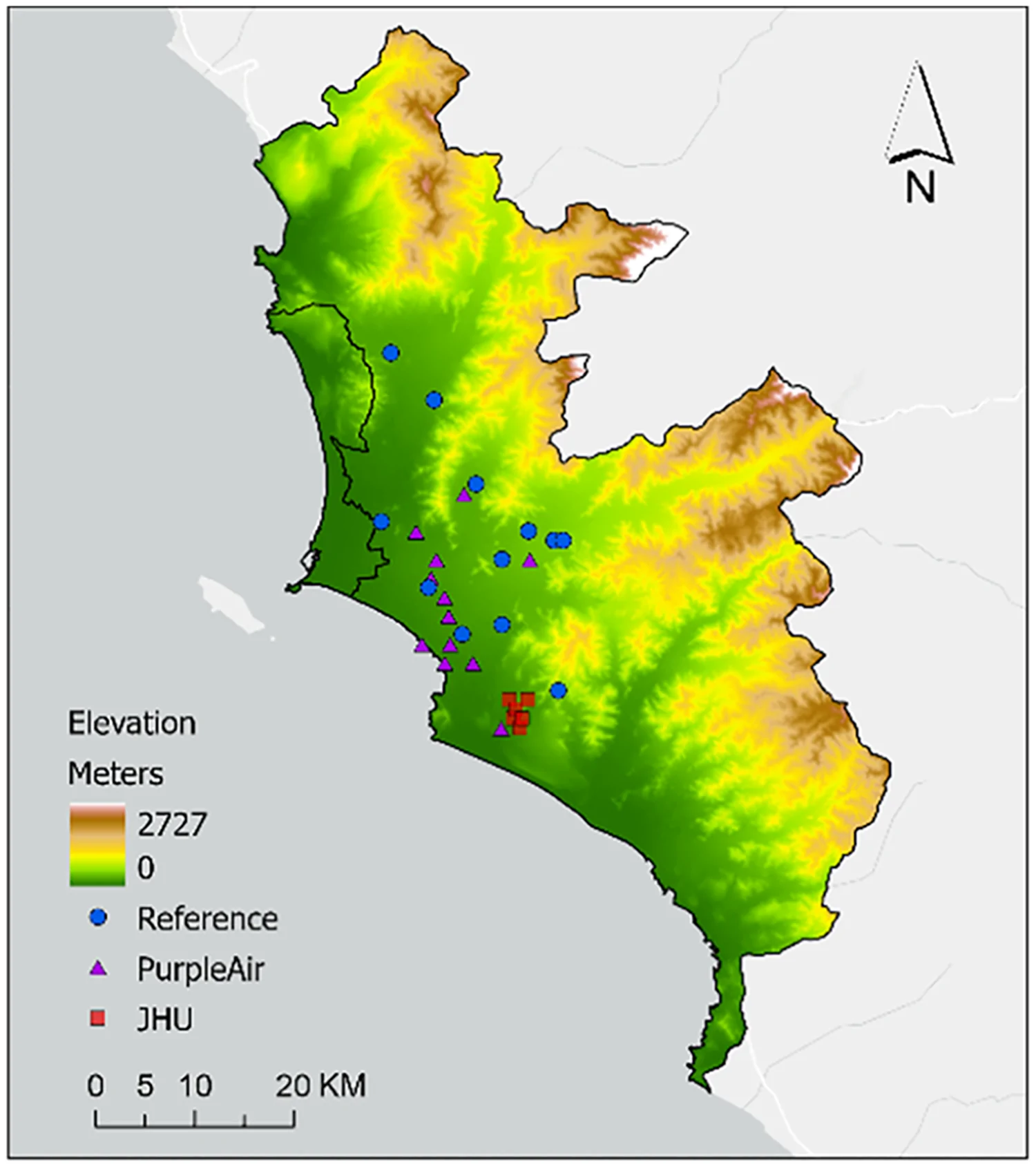
Study area of Lima, Peru, and monitoring locations of reference stations (blue circle), mobile monitors (JHU, red squares), and PurpleAir (purple triangles).

**Figure 2.**
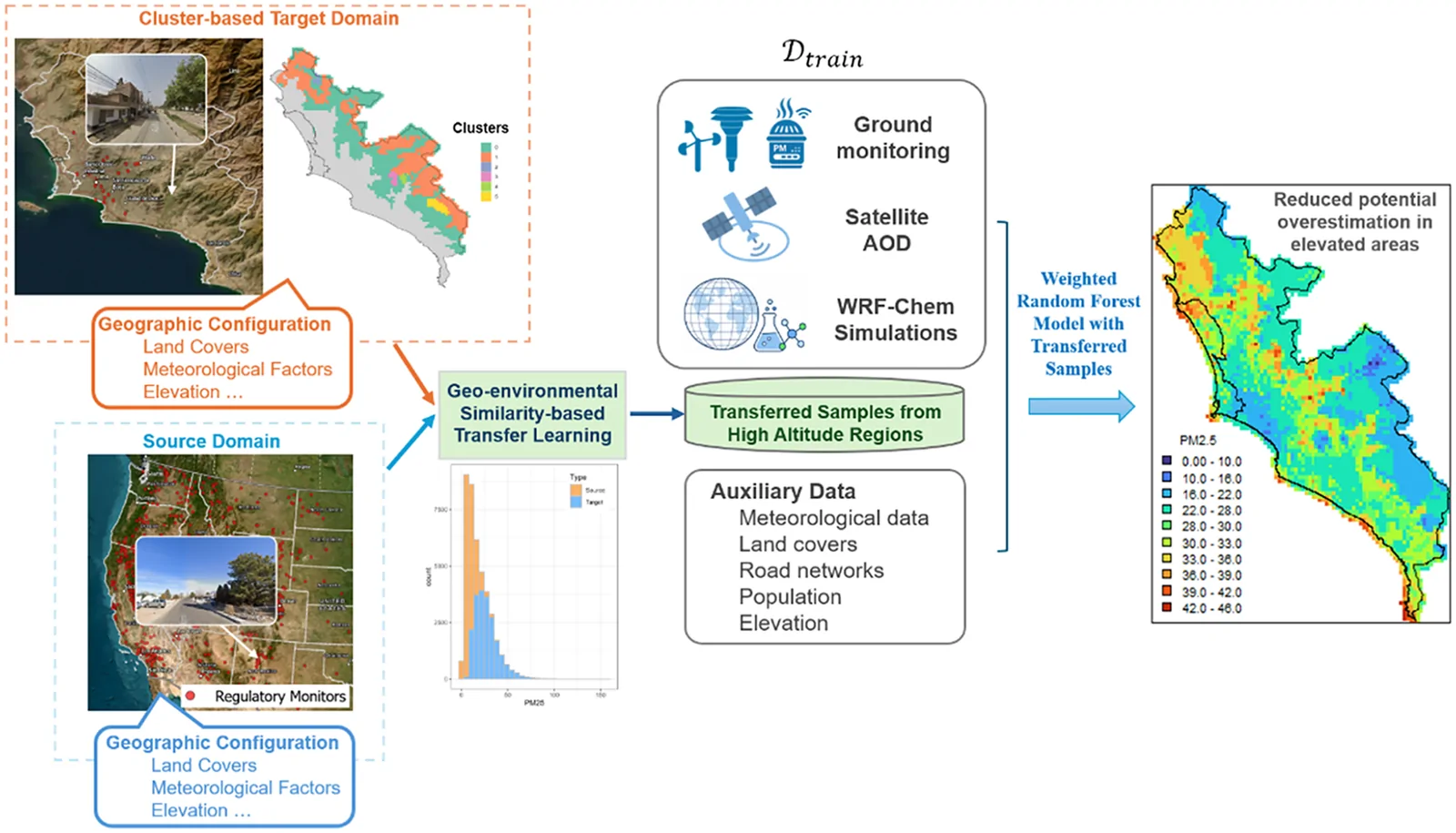
Simplified workflow for geographic similarity-based transfer learning approach.

**Figure 3.**
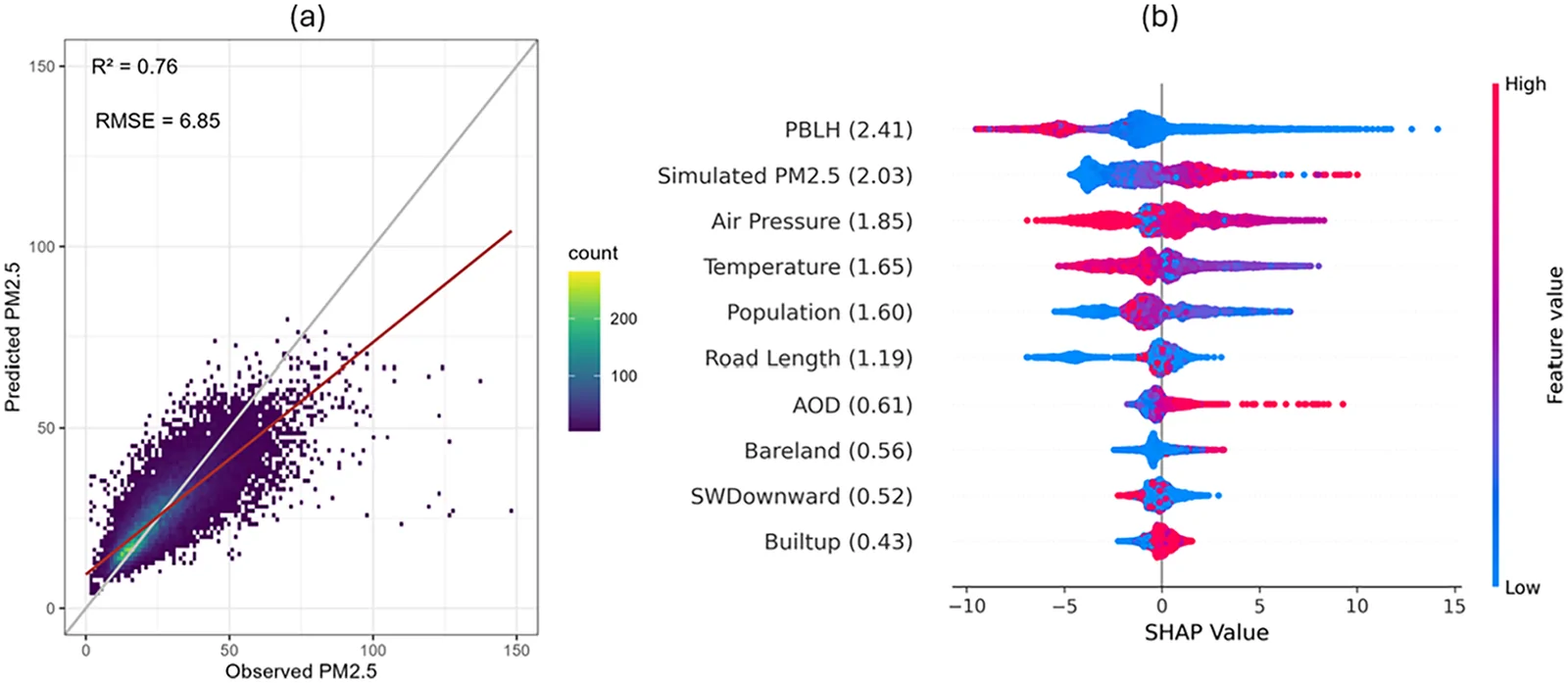
(a) 10-fold CV and (b) 10 most important variables ranked by SHAP for the TF_RF PM_2.5_ prediction model.

**Figure 4.**
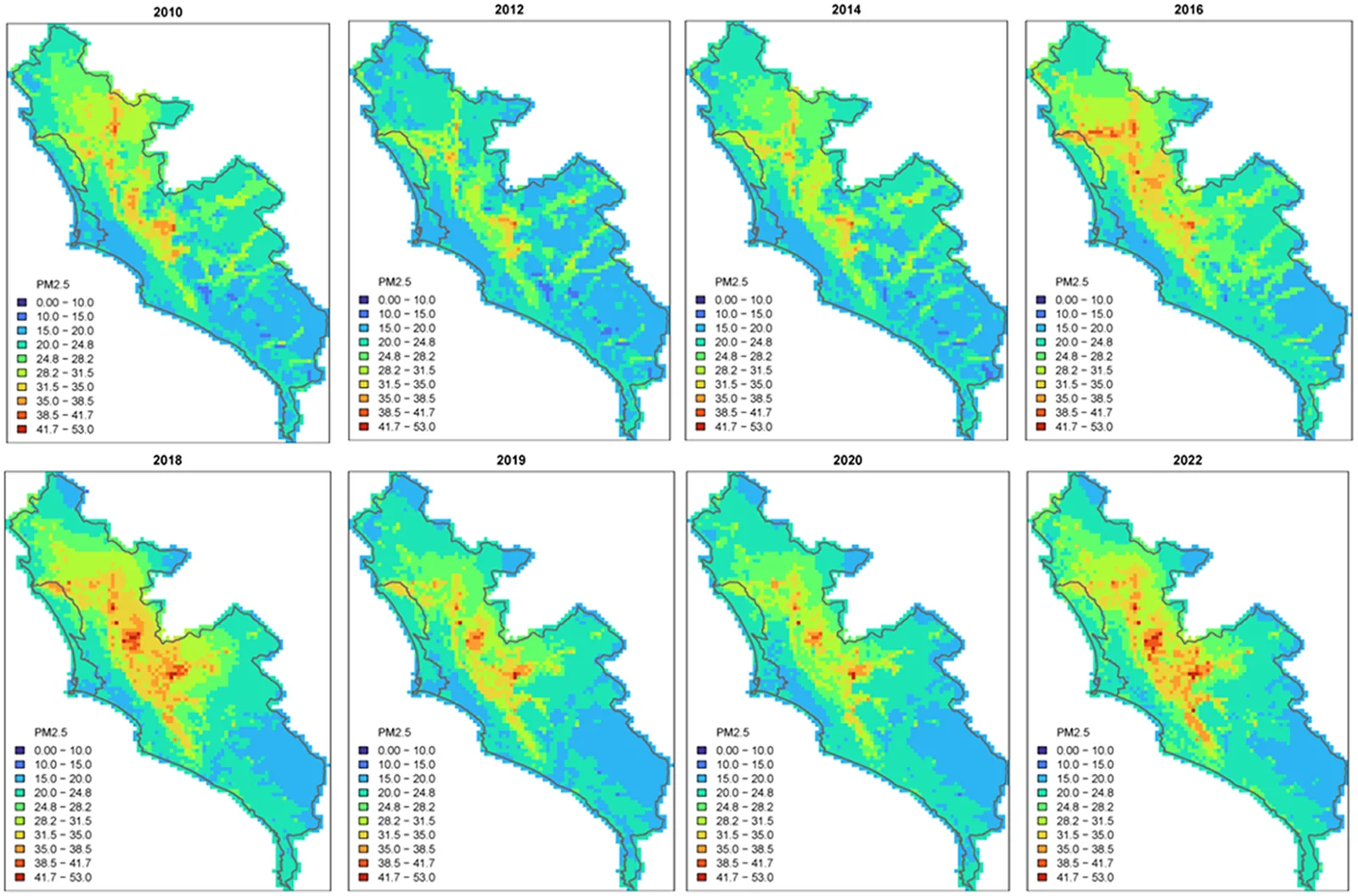
Annual PM_2.5_ distributions from 2010 to 2023 in Lima, Peru.

**Figure 5.**
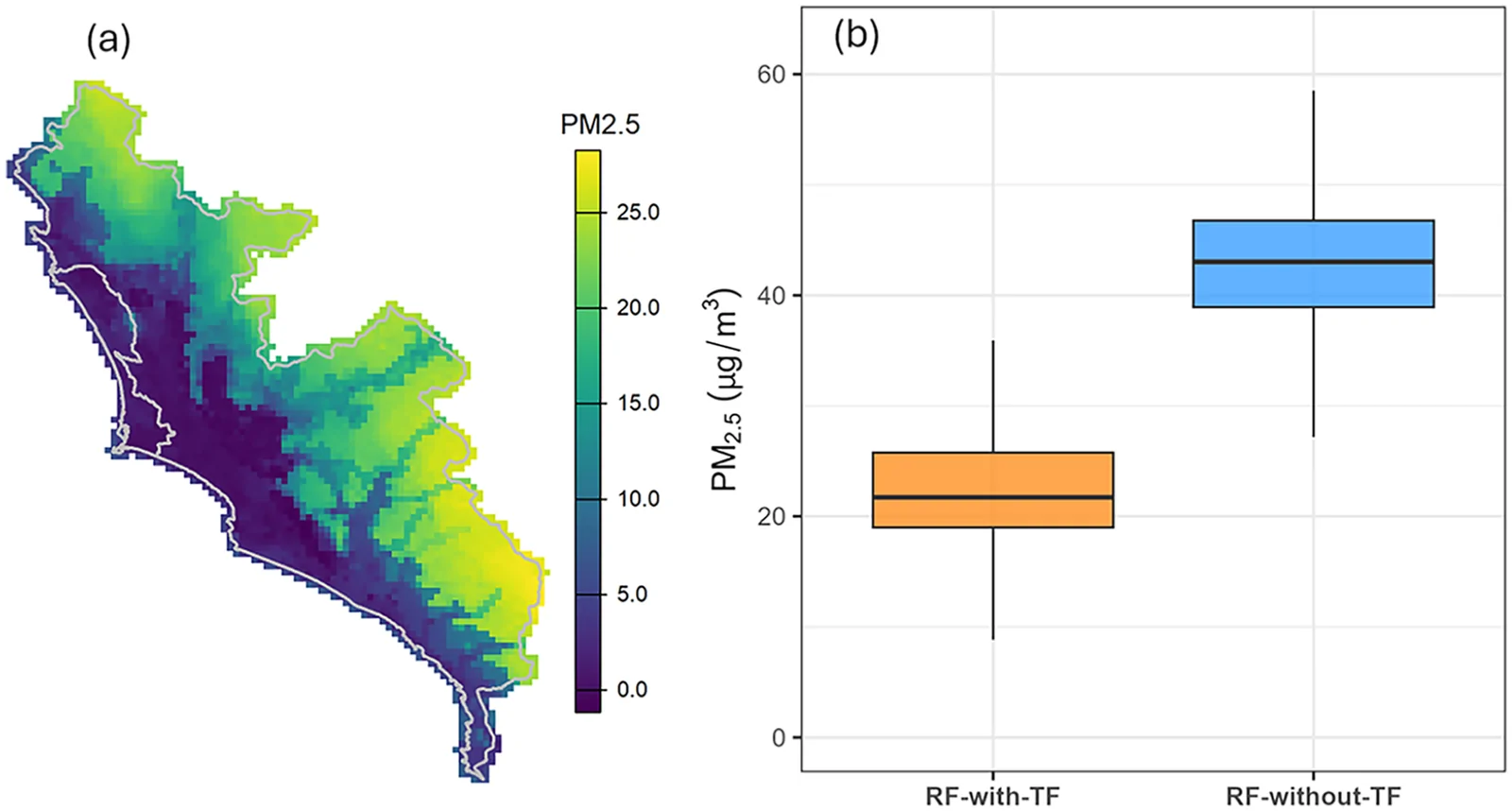
(a) Averaged prediction difference map from 2010 to 2023 (TL_RF–Regular_RF). (b) Boxplots of PM_2.5_ predictions of TL_RF (orange) vs Regular_RF (blue) over areas with elevation ≥370 m.
