# Supplementary material for: A Hybrid Transfer Learning Approach for Ambient PM2.5 Exposure Modeling in LMIC Contexts: A Case Study from Lima, Peru
Source: ACS EST Air. Author manuscript; Available in PMC 2026 Oct 15. (PMC13663721; doi:10.1021/acsestair.6c00125)
Supplement: Supplementary file 1 [file NIHMS2215511-supplement-Supporting_Information.pdf]

Supporting Information

A hybrid transfer learning approach for ambient $PM_{2.5}$ exposure modeling in LMIC contexts: A case study from Lima, Peru

Qiang Pu[a], Qingyang Zhu[b], Elvis Medina[c], Alan Llacza[c], Bryan Vu[d], Jianzhao Bi[e], Kenan Li[a], Laura Nicolaou[f,g,h], William Checkley[f,h], Stella Hartinger[i], Kyle Steenland[b], Yang Liu[b, *]

[a] College for Public Health and Social Justice, Saint Louis University, St. Louis, Missouri 63103, United States
[b] Gangarosa Department of Environmental Health, Rollins School of Public Health, Emory University, Atlanta, Georgia 30322, United States
[c] National Meteorological and Hydrological Service of Peru, Jr. Cahuide 785, Jesús María, Lima 15072, Peru
[d] Department of Environmental Health, Harvard TH Chan School of Public Health, Boston, Massachusetts 02115, United States
[e] Department of Environmental and Occupational Health Sciences, University of Washington, Seattle, Washington 98195, United States
[f] Division of Pulmonary and Critical Care, School of Medicine, Johns Hopkins University, Baltimore, Maryland 21205, United States
[g] Department of Environmental Health and Engineering, Bloomberg School of Public Health, Johns Hopkins University, Baltimore, Maryland 21205, United States
[h] Center for Global Non-Communicable Disease Research and Training, School of Medicine, Johns Hopkins University, Baltimore, Maryland 21205, United States
[i] Centro Latino Americano de Excelencia en Cambio Climático y Salud, Universidad Peruana Cayetano Heredia, Lima 15102, Peru

*Corresponding author. E-mail: yang.liu@emory.edu

Section S1. Calculation of population-weighted $PM_{2.5}$ concentration

Because regional health data are reported at the district level (43 in Lima city and 6 in Callao), we also calculate the population-weighted $PM_{2.5}$ concentrations for each district to investigate the potential impact of incorporating transfer-learning approach in exposure assessment. The population-weighted $PM_{2.5}$ can be expressed as:

$$Y_{d,j}^{Pop} = \sum_{i=1}^{N_d} \left(Pop_{i,j} \times \hat{Y}_{i,j}\right) \Big/ \sum_{i=1}^{N_d} Pop_{i,j}$$

where, $Y_{d,j}^{Pop}$ represents the daily population-weighted $PM_{2.5}$ concentration for each district d for date j. $\hat{Y}_{i,j}$ denotes daily predicted $PM_{2.5}$ at grid cell i and date j that falls within district d ($N_d = 49$). $Pop_{i,j}$ is the total population count at grid cell i and date j within district d for the year that j belongs to, which is based on the annual gridded population data.

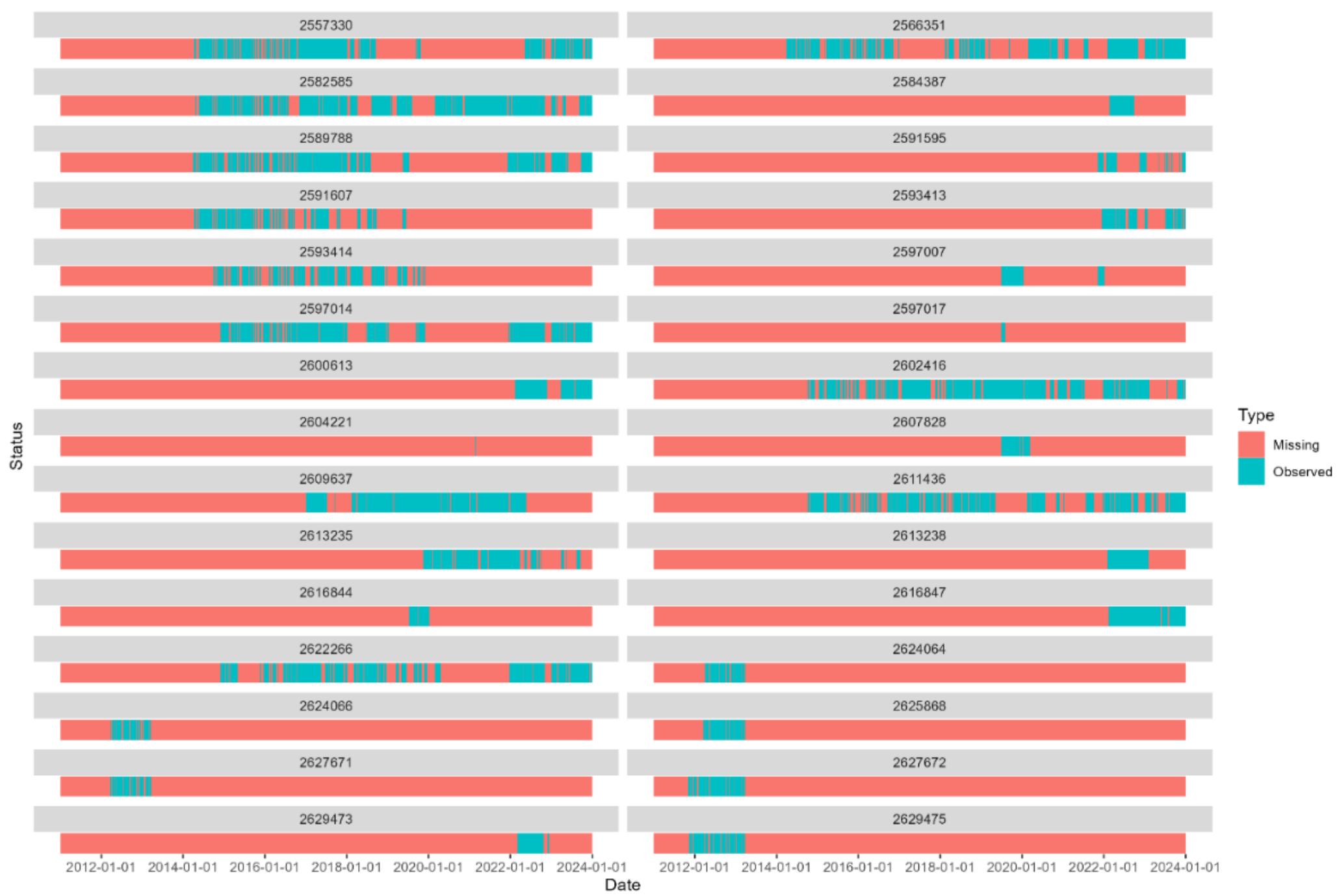

Fig. S1 Data completeness from ground monitors from 2010 to 2023.

Table S1 Hyperparameters for random forest model.

Parameter	Selected value (value range)
Number of trees	150 (100-800, by 50)
Number of variables selected (mtry)	5 (5-15, by 1)
Sample rate	0.8 (0.5-1.0, by 0.05)
Minimum node size	11 (4-12, by 1)

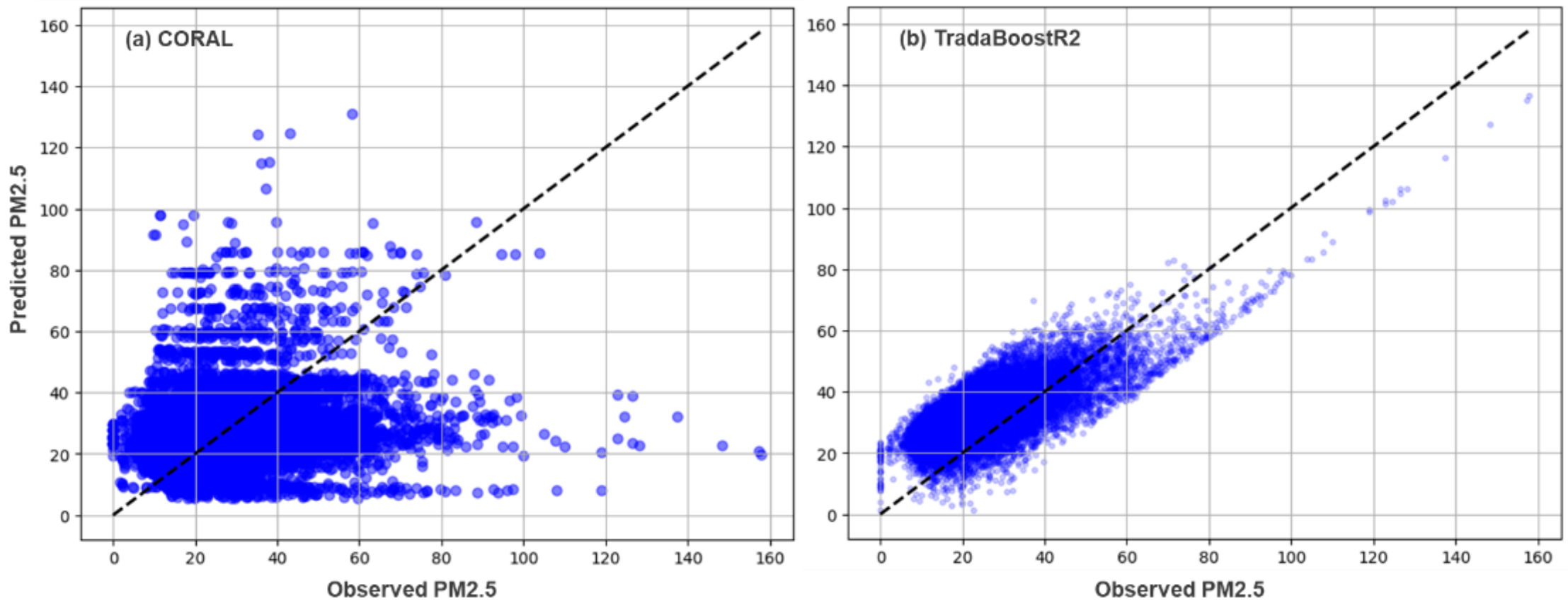

Fig. S2 Performance comparison of transfer-learning models: (a) CORAL and (b) TradaBoostR2.

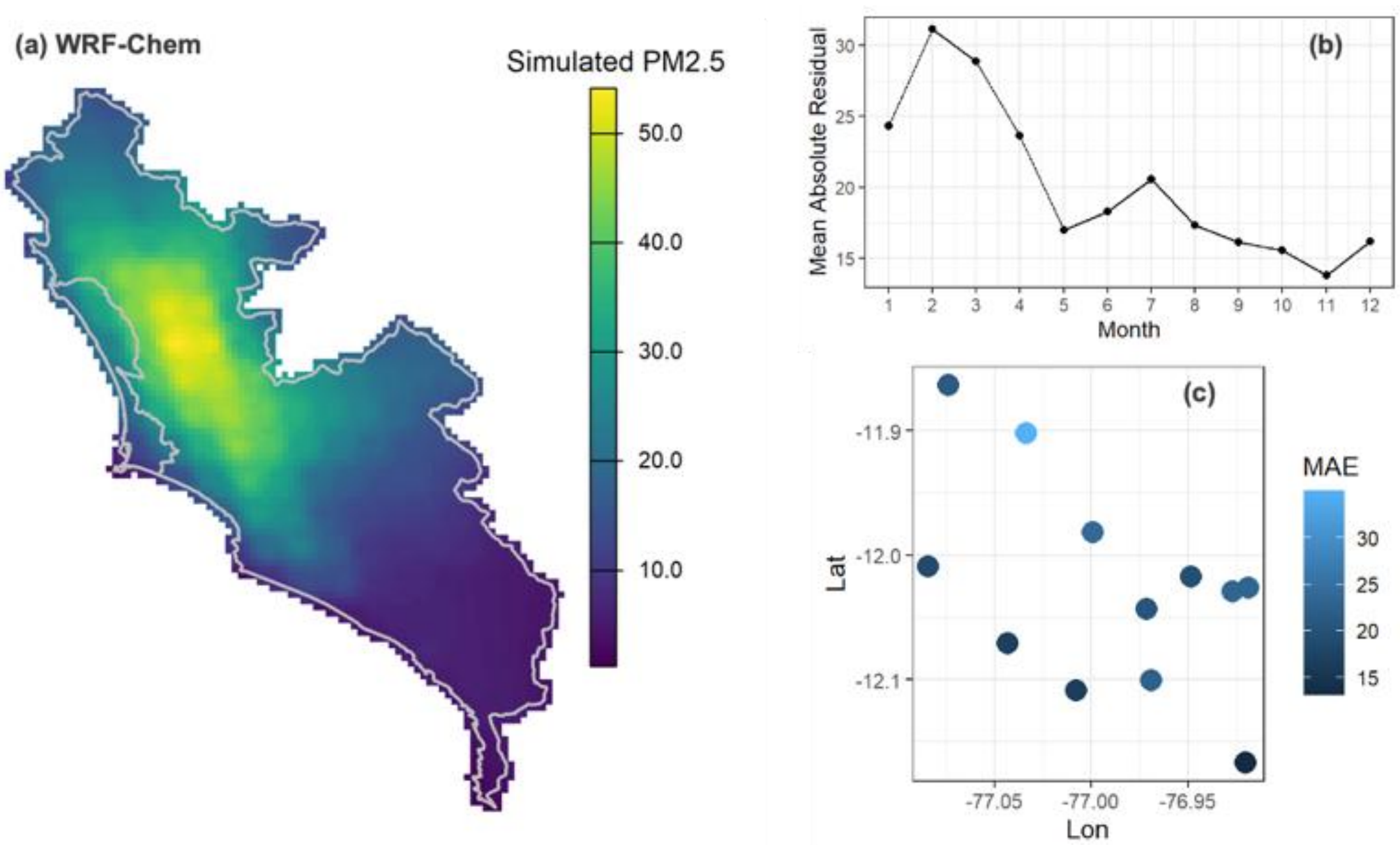

Fig. S3 (a) Spatial distributions of WRF_Chem simulated $PM_{2.5}$ concentration 2010 to 2023; (b) monthly variation in mean absolute error (MAE) against reference-grade observations; (c) overall spatial distribution of MAE against reference-grade observations.

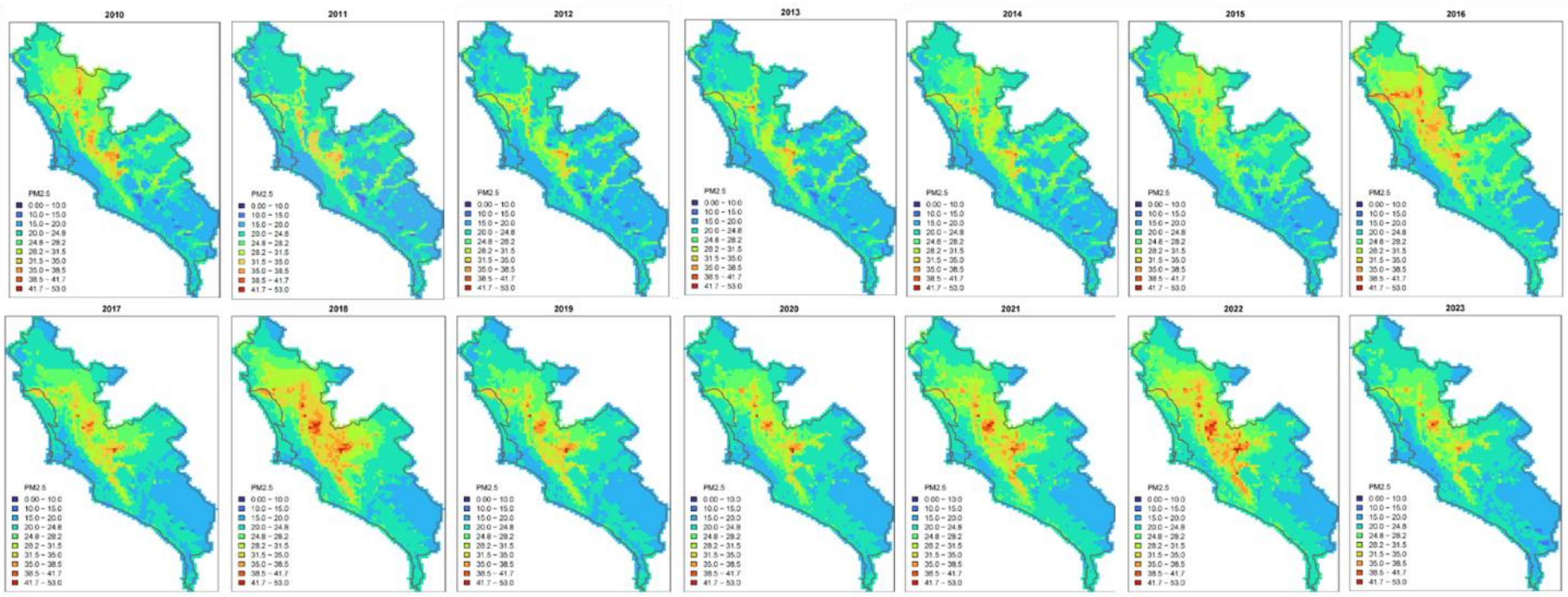

Fig. S4 Annual $PM_{2.5}$ distributions from 2010 to 2023 in Lima.

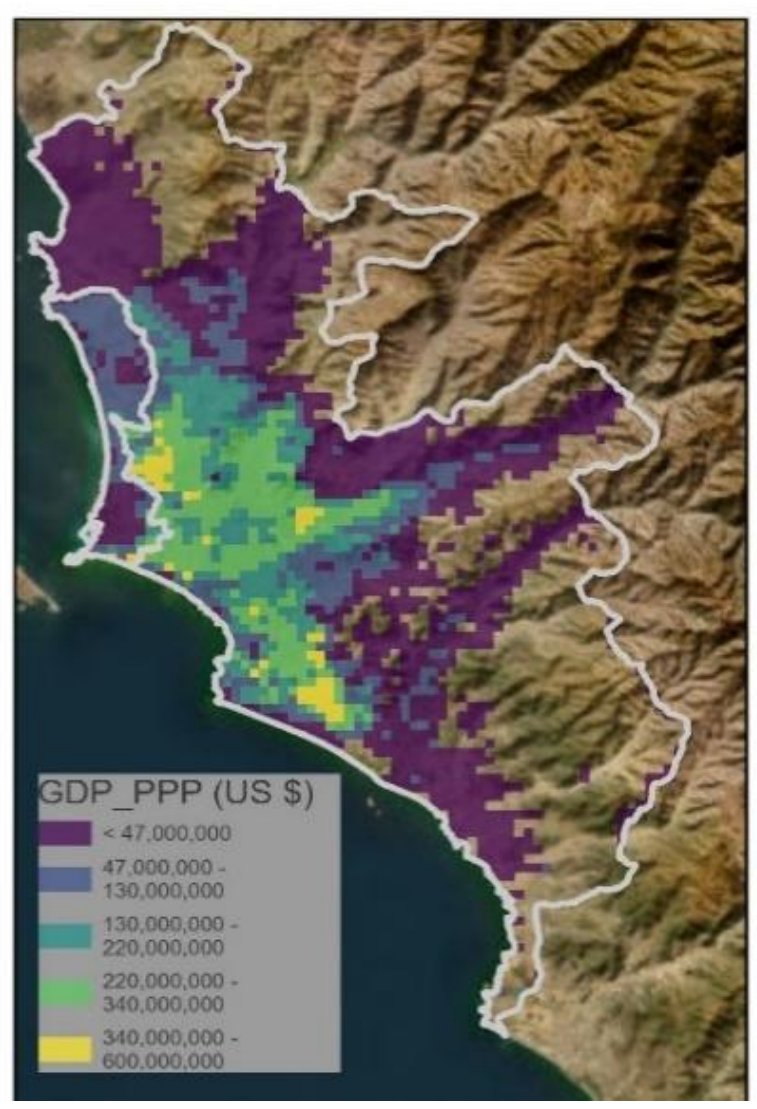

Fig. S5 Estimated gross domestic product based on purchasing power parity (GDP_PPP) by (Kummu et al., 2025)

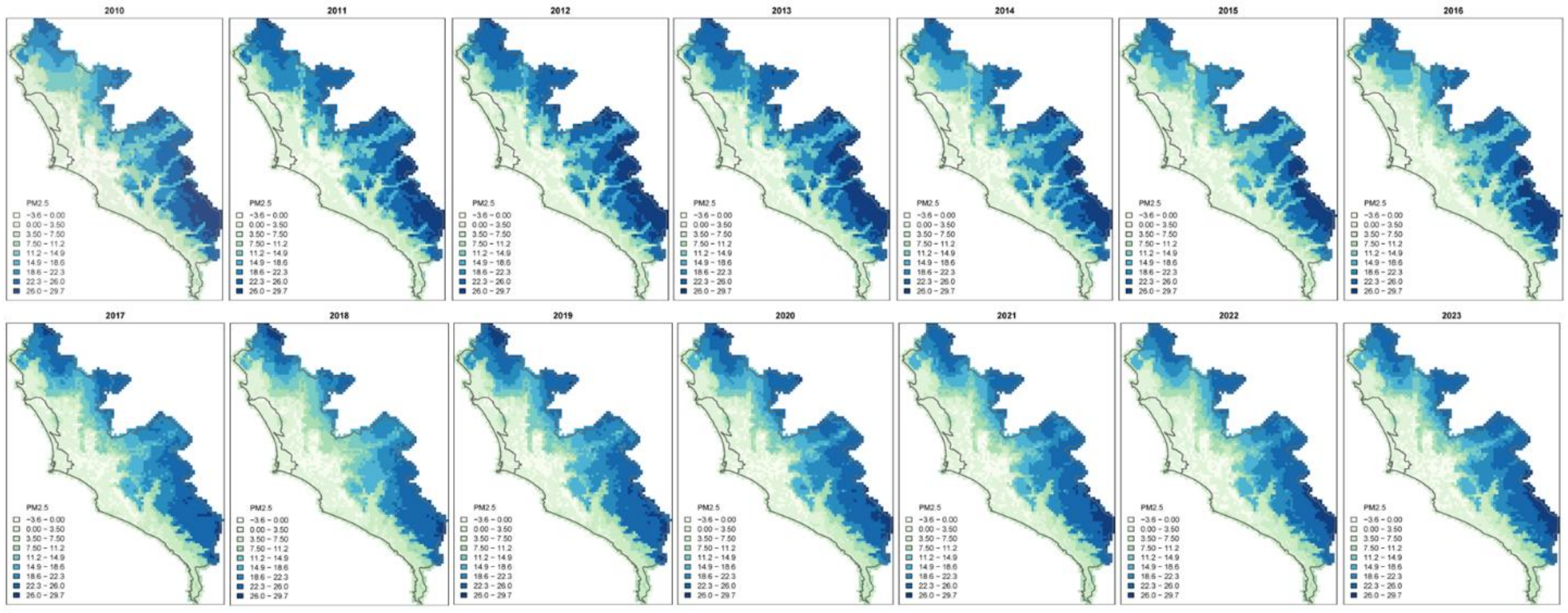

Fig. S6 Spatial distributions of annual mean differences in $PM_{2.5}$ predictions (Regular_RF − TL_RF), from 2010 to 2023.

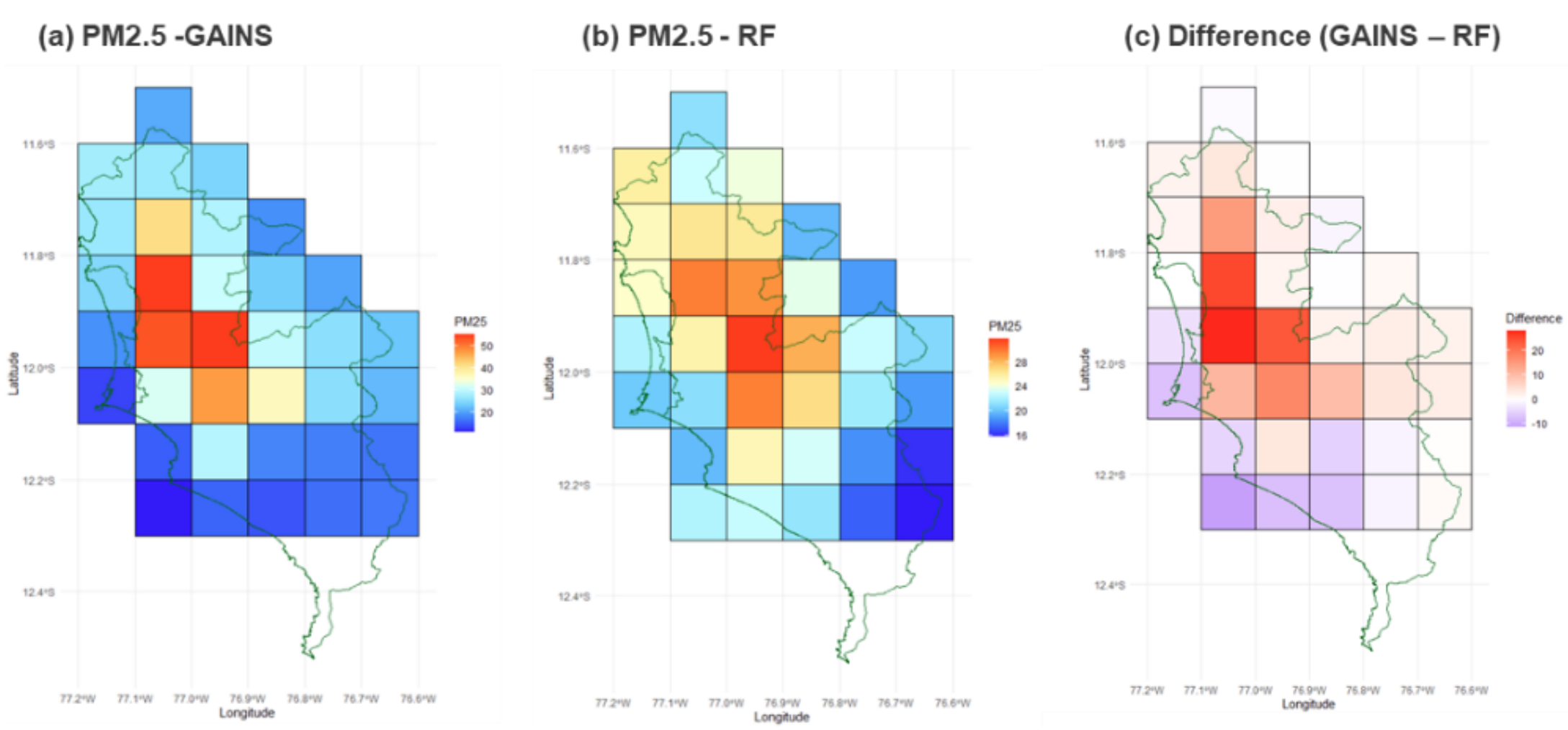

Fig. S7 $PM_{2.5}$ predictions from (a) GAINS (the Greenhouse gas - Air pollution Interactions and Synergies) and (b) transfer-learning based RF model prediction for year of 2020. Note: the GAINS model covers 36 grids at a resolution of 0.1° × 0.1° across Lima. The correlation coefficient between GAINS and RF was 0.78, R^2 of 0.61, and an absolute difference of 6.12 μg/m^3.

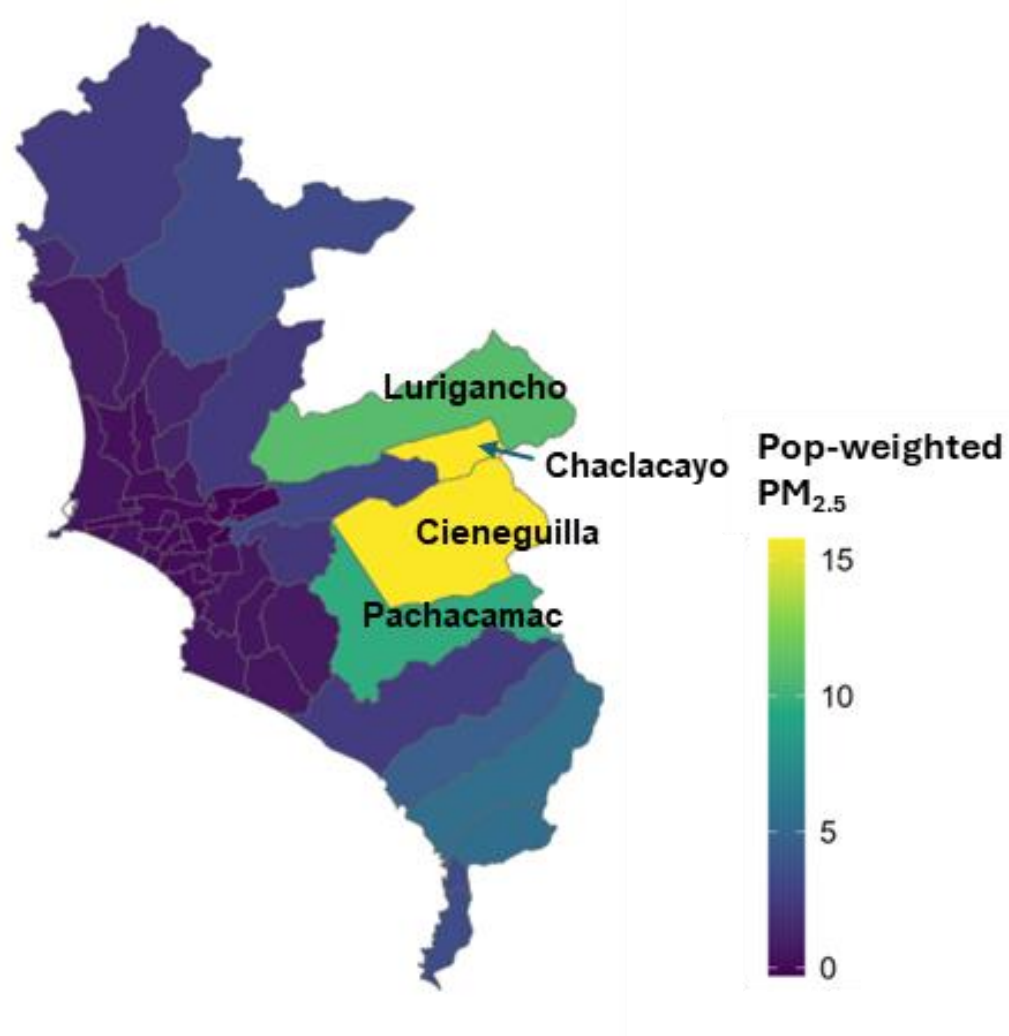

Fig. S8 Differences in population-weighted $PM_{2.5}$ in Lima from 2010 to 2023 (Regular_RF – TL_RF).

References

Kummu, M., Kosonen, M., & Masoumzadeh Sayyar, S. (2025). Downscaled gridded global dataset for gross domestic product (GDP) per capita PPP over 1990–2022. *Scientific Data*, *12*(1), 178. https://doi.org/10.1038/s41597-025-04487-x
